# Analysis of the differential transcriptome expression profiles during prenatal muscle tissue development in Diqing Tibetan pigs

**DOI:** 10.3389/fvets.2025.1584236

**Published:** 2025-10-31

**Authors:** Shuyuan Luo, Ying Bai, Xinpeng Li, Siqi Jin, Dawei Yan, Xinxing Dong

**Affiliations:** ^1^College of Animal Science and Technology, Yunnan Agricultural University, Kunming, China; ^2^School of Life Sciences and Food Engineering, Hebei University of Engineering, Handan, Hebei, China

**Keywords:** Diqing Tibetan pig, myofiber development, embryonic stages, transcriptome analysis, functional genes

## Abstract

Since the number of muscle fibers in pigs is largely fixed after birth, the formation of muscle fibers during the embryonic stage plays a crucial role in determining postnatal growth performance and meat production potential. In this study, we used large Diqing Tibetan pigs (LTP) and small Diqing Tibetan pigs (STP), which show significant differences in postnatal growth rate and meat yield, as research models. We employed RNA-seq for transcriptome sequencing and applied differential expression analysis combined with weighted gene co-expression network analysis (WGCNA) to compare their gene expression profiles and identify potential regulatory differences during key stages of embryonic muscle development. Longissimus dorsi muscle samples were collected from both groups at three critical developmental stages—embryonic day 55 (E55), embryonic day 75 (E75), and at birth (D0)—for transcriptome sequencing. Differential expression analysis revealed that the higher meat yield observed in LTP compared with STP may be attributed to a stronger capacity for secondary muscle fiber formation during the embryonic stage. Furthermore, WGCNA identified candidate genes that may specifically regulate muscle development in LTP across the three key developmental stages. These findings provide valuable insights into the molecular regulatory networks underlying muscle development and growth potential in Diqing Tibetan pigs.

## 1 Introduction

Skeletal muscle development is a complex process involving the formation of muscle fibers during embryonic development and hypertrophy after birth (1). Myofibers originate from myoblasts that proliferate and fuse to form myotubes, which then differentiate into mature myofibers. In pigs, the formation of skeletal muscle during embryonic development involves two key phases: the establishment of primary myofibers and the emergence of secondary myofibers. The formation of primary myofiber occurs during early gestation (from day 35 to day 55), when precursor myogenic cells fuse to form myotubes (2). Following the formation of primary fibers, secondary fiber development occurs from day 55 to day 90, during which myogenic cells rapidly proliferate and differentiate, utilizing the primary fibers as a scaffold for the formation of secondary fibers (3). Following birth, the number of muscle fibers in pigs remains stable, with the primary change being an increase in fiber size (4). Consequently, the embryonic stage of skeletal muscle development is vital. Elucidating the genetic mechanisms that regulate this process, particularly during early development, is essential for improving pork production efficiency.

The Diqing Tibetan pig is an excellent local breed primarily distributed in the Diqing Tibetan Autonomous Prefecture of Yunnan Province, China. As a typical plateau breed, Diqing pigs exhibit low oxygen tolerance (5–8) and possess outstanding meat quality traits (9–13). Based on body size differences, Diqing Tibetan pigs can be categorized into large, medium, and small types. Adult large pigs can weigh between 70 and 150 kg, with an average daily gain of 200–250 grams during the fattening period; small pigs typically weigh between 45 and 55 kg, gaining 100–120 grams daily during the same phase (14–16). These body size differences not only affect the growth rate and meat quality characteristics of the pigs but also provide important material for analyzing the functional genes involved in variations in muscle fiber development within the breed. Currently, research on Diqing Tibetan pigs mainly focuses on various aspects, including origin and domestication (17), genetic diversity (18–20), hybrid utilization (21, 22), growth characteristics (23, 24), high-altitude adaptation (25), fat deposition (26–29), and postnatal muscle development (30–32). Despite the existing studies covering multiple areas, research on the differential expression of genes involved in muscle development during the embryonic stages of large and small Diqing Tibetan pigs is still limited. In this study, we utilized both large and small Diqing Tibetan Pigs, which exhibit marked differences in growth rate, and lean meat percentage. Our objective was to identify key genes that influence muscle fiber development at various stages across different body types. These findings will provide a valuable foundation for genetic improvement and effective breeding strategies.

## 2 Materials and methods

### 2.1 Ethical statement

All experimental procedures in this study received approval from The Ethics Committee of Life Sciences, Yunnan Agricultural University (approval number: 202207003).

### 2.2 Sample collection

The experimental subjects included large Diqing Tibetan pigs (LTP) and small Diqing Tibetan pigs (STP), all housed and fed under uniform conditions at the Lvyuan Agricultural Professional Cooperative in Shangri-La City, Yunnan Province, China. Our research team previously established two distinct lineages characterized by large and small body types through a long-term selective breeding program focused on growth rate and adult body weight differences. At 6 months of age, large Diqing Tibetan pigs had an average weight of 59.33 ± 5.77 kg, while small Diqing Tibetan pigs averaged 28.00 ± 2.00 kg. Purebred sows from each breed were synchronized in estrus and mated with purebred boars of the same breed. Three embryos or piglets at the identical developmental stage were all derived from the same maternal sow: embryonic day 55 (E55), embryonic day 75 (E75), and immediately after birth (D0), designated as LTP-E55, LTP-E75, LTP-D0 (and similarly for STP). Three fetuses or piglets from every group were randomly chosen for longissimus dorsi (LD) muscle sampling, subsequently flash-frozen in liquid nitrogen and preserved at −80 °C in the laboratory.

### 2.3 Tissue total RNA extraction and sequencing

Total RNA extraction from tissue samples was performed using the Trizol method, and nucleic acid integrity was verified via agarose gel electrophoresis. The optical density (OD) values of the nucleic acids were measured using a NanoDrop spectrophotometer to assess their purity. RNA integrity number (RIN) assessment was performed using the Agilent 2100 Bioanalyzer to evaluate quality. Following quality control, the RNA samples were submitted to Genedenovo Biotechnology Co., Ltd. (Guangzhou, China) for paired-end sequencing. The cDNA library was sequenced on the Illumina NovaSeq 6000 platform with 6 Gb of raw reads per biological replicate.

### 2.4 Raw data quality control and alignment

Raw sequencing data (Raw reads) were first processed with the FASTP tool (version 0.23.4) (33) for quality control. In this step, reads with adapters or poly-N sequences, over 10% unknown nucleotides, and more than 50% low-quality bases (Q ≤ 10) were eliminated. This quality control procedure produced high-quality reads (Clean reads), ensuring accuracy in subsequent analyses. The Clean reads were aligned to the reference genome of the pig (Sscrofa11.1) with HISAT2 (version 2.2.1) (34), and alignment rates were subsequently calculated. Finally, transcripts were assembled and quantified using STRINGTIE (version 2.2.3) (35), producing a raw expression matrix (Counts) for each gene in each sample.

### 2.5 Identification of differentially expressed genes and functional enrichment analysis

To account for sequencing depth and adjust for gene length effects, raw read counts were normalized to gene length corrected trimmed mean of M-values (GeTMM) (36) for all genes in each sample. The GeTMM normalized matrix encompassing all genes across all samples was subjected to principal component analysis (PCA) using the FactoMineR package (version 2.11) (37).

Following PCA confirmation of high-quality sequencing data and reliable sample grouping, the analysis was performed using the edgeR package (version 3.40.2) (38), differentially expressed genes (DEGs) were identified based on the GeTMM of genes, with statistically significant DEGs defined by dual thresholds of absolute log_2_ (Fold Change) > 1.0 and adjusted *p*-value (FDR) < 0.05. Enrichment analysis for Gene Ontology (GO) was performed on genes from significantly correlated modules by KOBAS online database^1^ (39).

### 2.6 Weighted gene co-expression network analysis

Using the GeTMM values of each gene across all samples, we performed weighted gene co-expression network analysis (WGCNA) in R using the WGCNA package (version 1.73) (40). The weighted adjacency matrix was constructed using the soft-thresholding power (β) of 9 to attain scale-free topology. The adjacency was transformed into a topological overlap matrix (TOM), and the corresponding dissimilarity (1-TOM) is calculated. The modules were identified by dynamic tree cutting and with a minimum module size of 50. A correlation of 70% (equivalent to a distance metric threshold of 0.3 in analytical pipelines) was used to merge similar modules. We conducted a correlation analysis between each module and the three developmental stages of different body types to identify relevant modules highly associated with LTP across these stages. Visualization was achieved through heatmaps, and modules with *p*-value < 0.05 were designated as significantly correlated modules for further analysis. A significance threshold of *p*-value < 0.05 was applied for enrichment analysis. We calculated kernel module eigengenes (KME) by assessing gene significance (GS) and module membership (MM) to identify hub genes within the modules.

### 2.7 Construction of the differentially expressed gene interaction network

We compared the identified DEGs with protein entries in the STRING database^2^ (41). Based on homology protein–protein interaction data, we created an interaction network for the identified DEGs, which was subsequently visualized using Cytoscape (version 3.10.1) (42). Nodes with the most neighbors were designated as key genes.

### 2.8 Validation by real-time quantitative PCR

The porcine *GAPDH* gene (43, 44) was employed as the internal reference. The total RNA for qPCR was derived from the RNA used in the RNA-seq experiments. The design of primers was conducted with Primer-BLAST,^3^ with the sequences provided in Table 1. Shenggong Biological Engineering (Shanghai) Co., Ltd., Kunming Branch (Kunming, China) synthesized all primers. In accordance with the manufacturer’s guidelines, qPCR was conducted using a SYBR Green qPCR kit (TIANGEN, China, FP205) on an FQD-96A real-time PCR detection system (Bioer, Hangzhou, Zhejiang, China). The reaction system had a total volume of 20 μL, which included 10 μL of 2 × SuperRreMixPlus, 0.6 μL of each forward and reverse primer (10 μM), 1 μL of cDNA, 0.5 μL of 50 × ROX Reference Dye, and 7.3 μL of RNase-free distilled water were added. The reaction program comprised an initial activation step at 95 °C for 15 min, followed by 40 cycles of denaturation at 95 °C for 10 s and annealing/extension at 60 °C for 20 s. A melting curve analysis was executed by incrementally increasing the temperature from 60 °C to 95 °C, with each sample evaluated in triplicate. Gene expression levels were normalized to *GAPDH*, and The 2^−ΔΔCt method was utilized to assess relative expression levels.

**Table 1:** Primers for real-time quantitative PCR.

Genes	Sequence (5′-3′)	Length
GAPDH	F: GACATCAAGAAGGTGGTGAAGCA	177
	R: GTCGTACCAGGAAATGAGCTTGA	
PDLIM3	F: CGGCCCAAACCTTTCATAATCC	153
	R: TAGGGGCCATCTTAGCAGCA	
CMYA5	F: GATGAAGAGGGCAAGACCAAGA	100
	R: TGGTCTCCCAGGTTATTCCAC	
ATP2A1	F: TTCAACGATCCTGTCCACGG	158
	R: GCGTTCTTCTTTGCCATCCG	
ACACB	F: CTGCACGGAAATGATCGCTG	213
	R: CCCTCATCTGGGTTTTCGCT	
CLCN1	F: GCACCGCCTGCTCTATC	211
	R: CACGACCACGTTGACTTTT	

## 3 Results

### 3.1 RNA-seq data quality assessment of LD muscle samples

**Figure 1:**
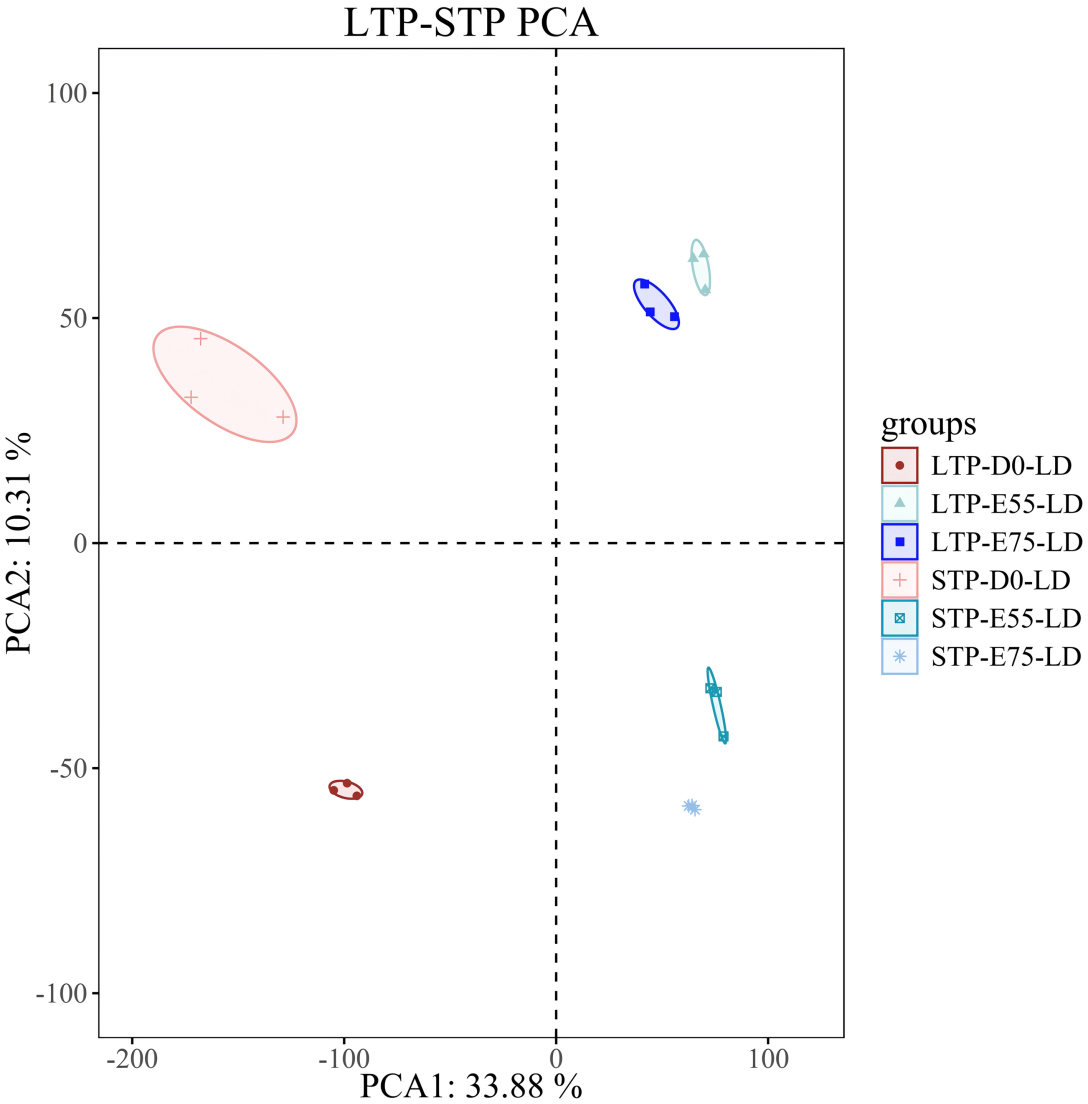
PCA plot of LTP and STP. Each dot color represents a different time point and each dot represents an individual pig.

The RNA-seq data showed that the LTP groups at three developmental stages generated an average of 38.37 M, 41.39 M, and 46.46 M raw reads, respectively. The STP groups produced an average of 38.21 M, 38.49 M, and 36.77 M raw reads. Following quality control, the clean read rate surpassed 98%, with over 95% of reads mapping to the porcine reference genome (Sscrofa 11.1), indicating high-quality alignment (Supplementary Table S1). Subsequently, PCA (Figure 1) demonstrated that biological replicates within each group clustered tightly, while clear separation was observed among different groups, occupying distinct positions. This confirms the high quality of the data and the reliability of sample grouping, supporting their suitability for downstream analysis.

### 3.2 DEGs regulating the LD muscle development

**Figure 2:**
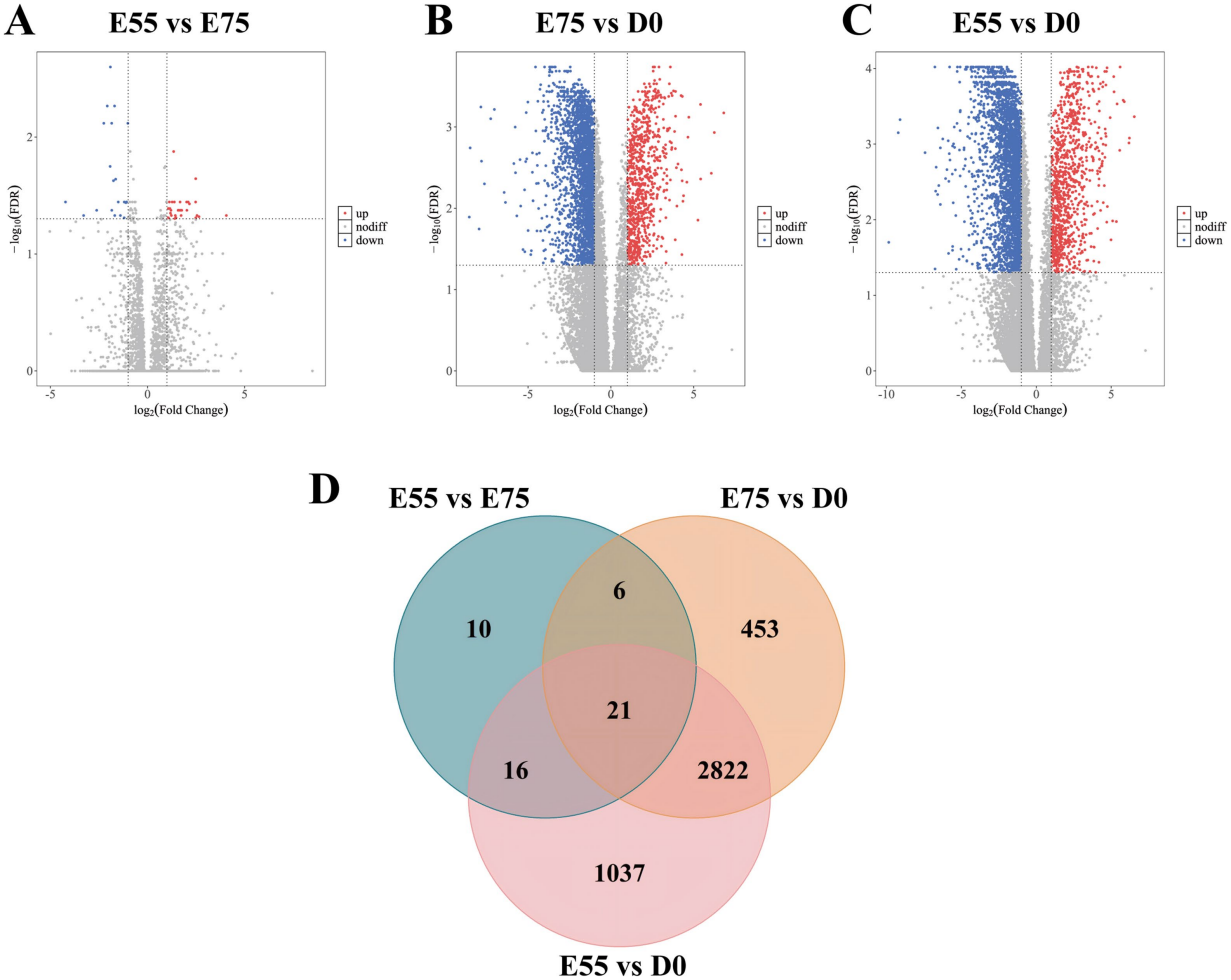
DEGs at three different developmental stages in LTP. **(A)** Volcano map of DEGs between E55 and E75. **(B)** Volcano map of DEGs between E75 and D0. **(C)** Volcano map of DEGs between E55 and D0. **(D)** Venn maps of DEGs at three different developmental stages in LTP. Each dot represents a gene, red dots represent genes up-regulated, blue dots represent genes down-regulated, and gray dots represent genes with non-significant differences. The sum of the numbers in each large circle represents the total number of DEGs expressed in the group, and the overlapping parts of the circles represent DEGs shared between groups.

**Figure 3:**
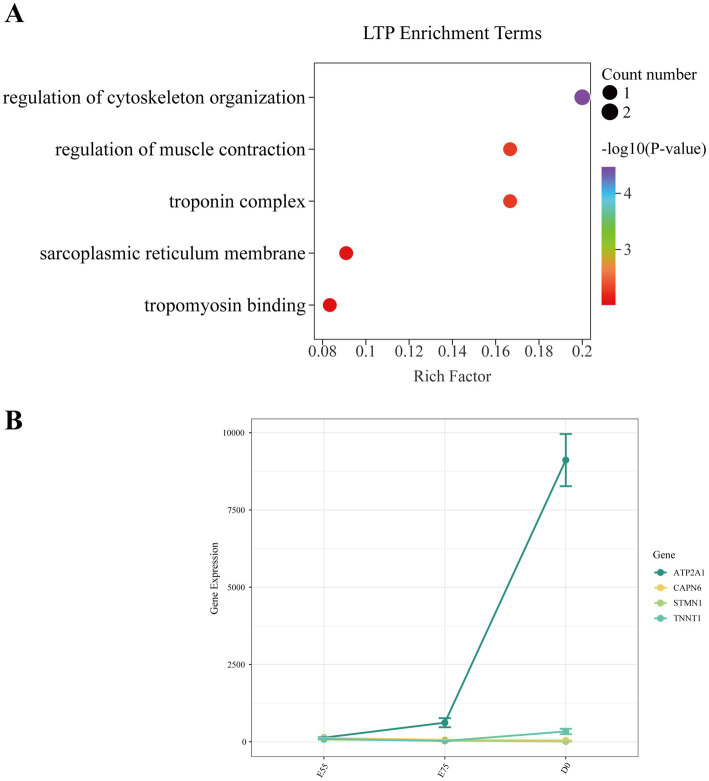
Expression changes of key pathway genes in LTP during development. **(A)** GO enrichment analysis of DEGs shared among the three comparisons. **(B)** Expression changes of key pathway genes during development.

During the critical developmental window from E55 to D0, the number of secondary myofibers formed in pigs directly determines postnatal meat yield. To identify functional genes regulating secondary myofiber formation and development, we performed longitudinal analysis of DEGs across developmental stages and identified consistently dynamically expressed candidates with relevant biological functions. In the LD muscle of LTP, across the different age groups, we identified 53 DEGs in the comparison of E75 vs. E55, 3,302 DEGs in D0 vs. E75, and 3,896 DEGs in D0 vs. E55 comparison (Figures 2A–C). A total of 21 DEGs were identified as shared among these three comparisons (Figure 2D; Supplementary Table S2). GO enrichment analysis revealed that these genes are significantly involved in key biological processes related to muscle growth and development (Figure 3A), including regulation of cytoskeleton organization (*CAPN6*, *STMN1*), troponin complex (*TNNT1*), and regulation of muscle contraction (*ATP2A1*). As shown in Figure 3B, the expression patterns of these genes across developmental stages exhibited distinct temporal dynamics: *ATP2A1* expression gradually increased over time, whereas *STMN1* and *CAPN6* showed progressive downregulation throughout development. In contrast, *TNNT1* expression displayed a biphasic trend, initially decreasing followed by a subsequent increase.

**Figure 4:**
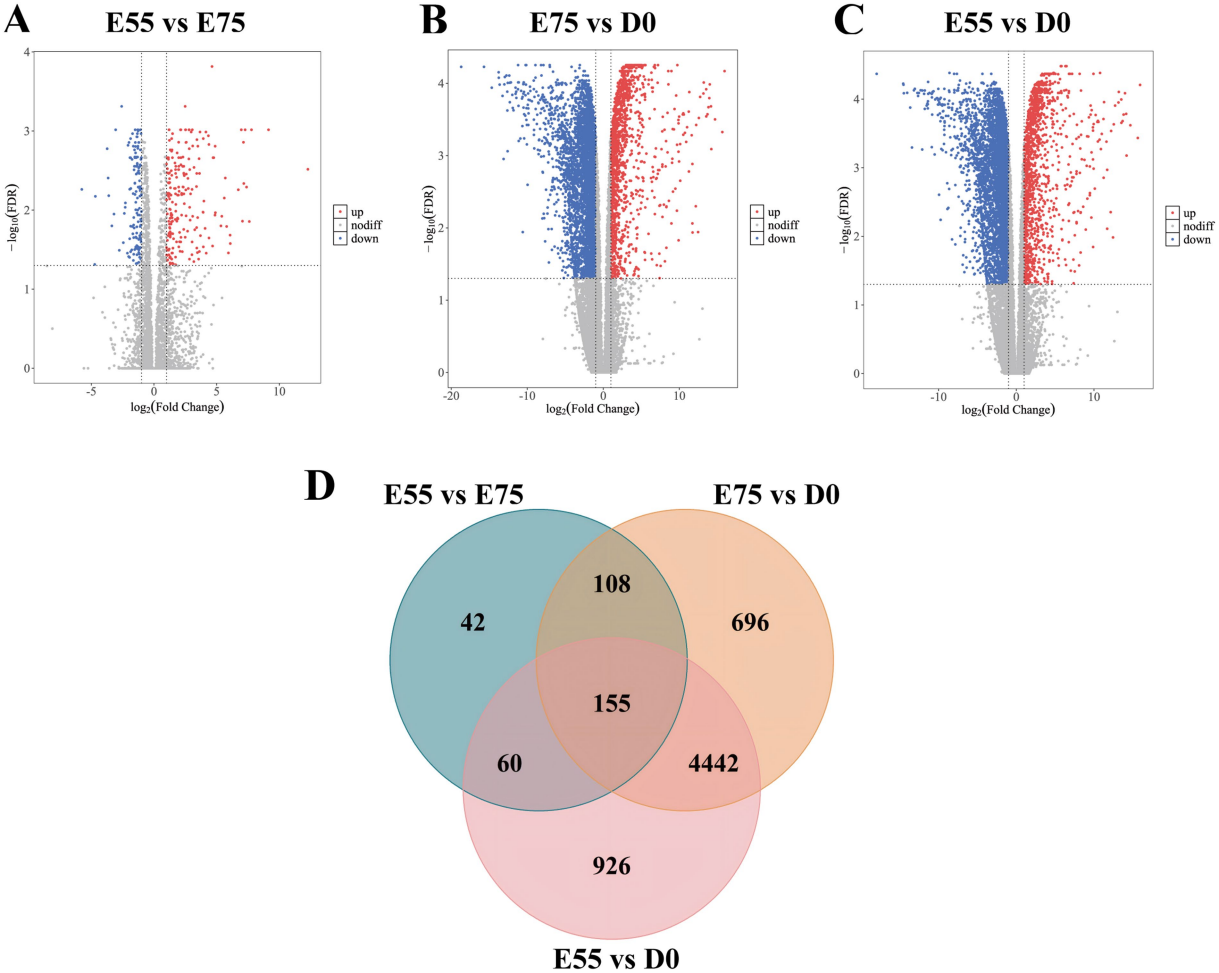
DEGs at three different developmental stages in STP. **(A)** Volcano map of DEGs between E55 and E75. **(B)** Volcano map of DEGs between E75 and D0. **(C)** Volcano map of DEGs between E55 and D0. **(D)** Venn maps of the number of DEGs at three different developmental stages in STP.

**Figure 5:**
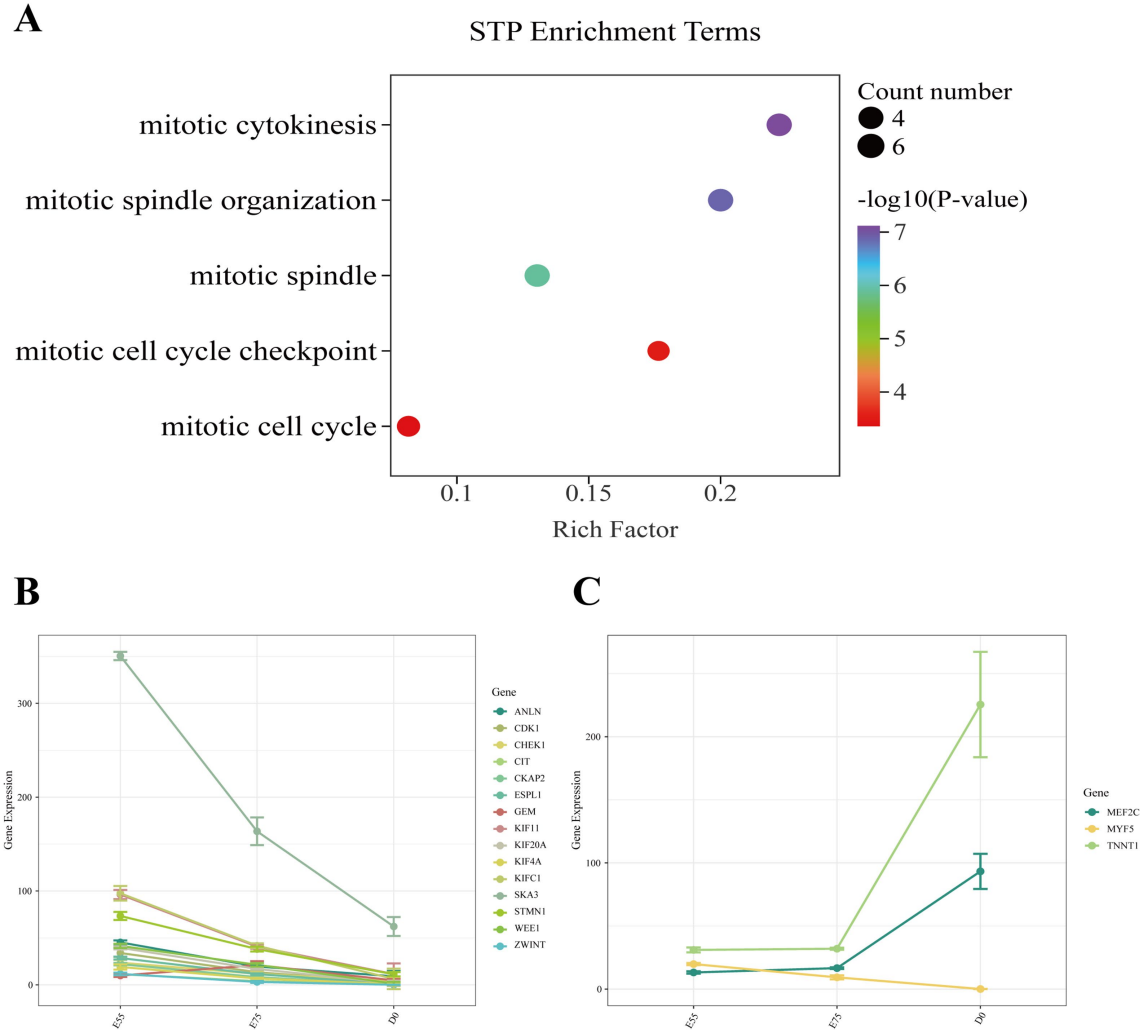
Expression changes of key pathway genes in STP during development. **(A)** GO enrichment analysis of DEGs shared among the three comparisons. **(B)** Expression changes of key pathway genes during development. **(C)** Expression changes of other key genes during development.

In STP, we identified 365 DEGs in the comparison of E75 vs. E55, 5,401 DEGs in D0 vs. E75, and 5,583 DEGs in D0 vs. E55 comparison (Figures 4A–C). A total of 155 DEGs were identified as shared among these three comparisons (Figure 4D; Supplementary Table S2). GO enrichment analysis revealed that these genes are significantly involved in key biological processes related to cell division, including the mitotic spindle (*CDK1*, *SKA3*, *ESPL1*, *KIF11*, *GEM*, and *KIFC1*), mitotic cytokinesis (*STMN1*, *ANLN*, *KIF4A*, *CIT*, *CKAP2*, and *KIF20A*), and mitotic cell cycle checkpoint (*ZWINT*, *WEE1*, and *CHEK1*) (Figure 5A). Expression profiling across developmental stages demonstrated distinct temporal dynamics for these genes (Figure 5B): while *GEM* exhibited a biphasic expression pattern characterized by an initial increase followed by a decrease, all other DEGs showed progressive downregulation throughout development. In addition to these genes, we observed a gradual decrease in *MYF5* expression, whereas *MEF2C* and *TNNT1* displayed progressive upregulation over time (Figure 5C).

### 3.3 Gene co-expression networks across three developmental stages in LTP

#### 3.3.1 Co-expression modules associations with three developmental stages of LTP

**Figure 6:**
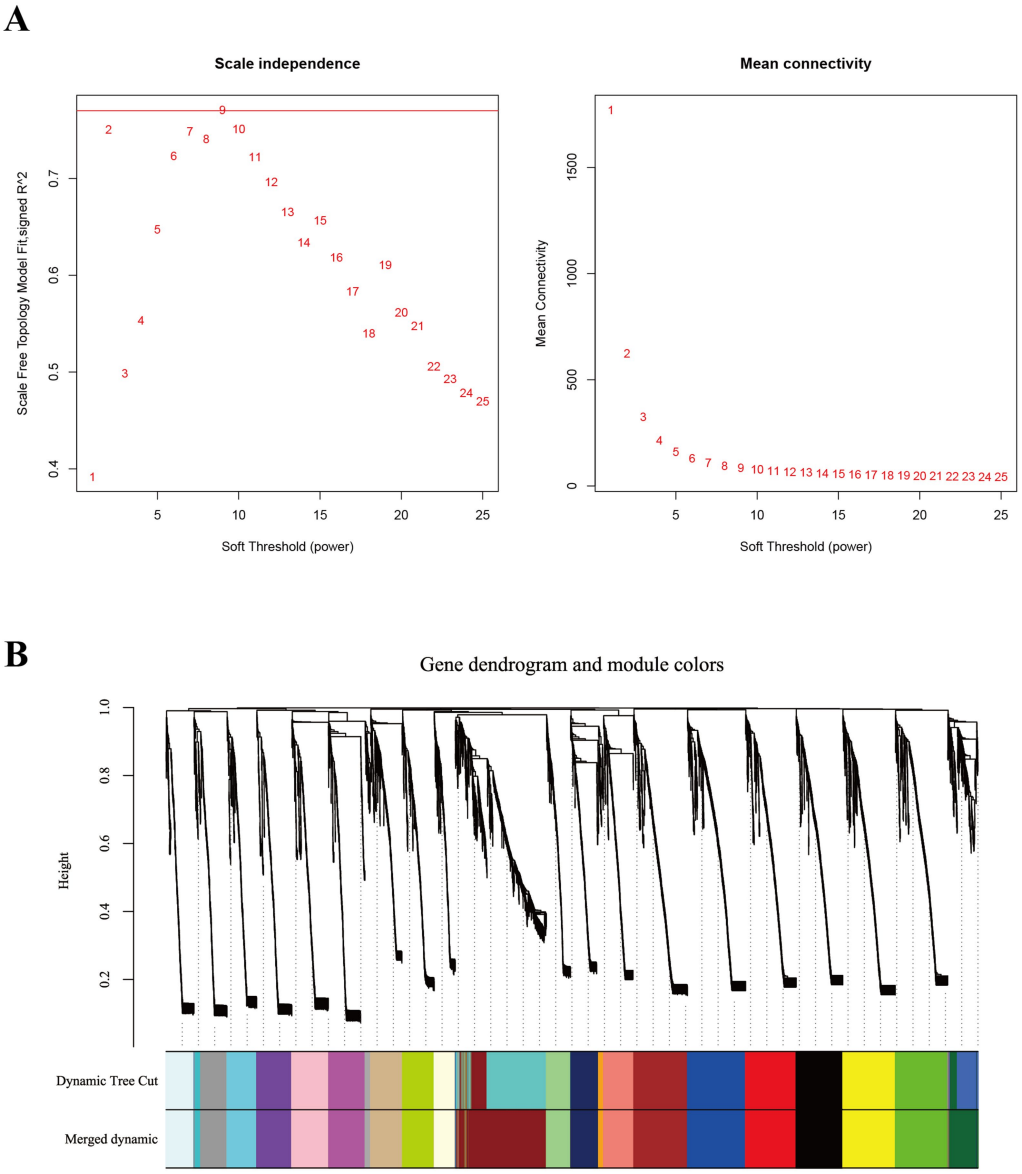
Identification of co-expression modules by WGCNA. **(A)** The determination of soft thresholding power. **(B)** The gene clustering dendrogram was obtained according to hierarchic clustering of adjacency based dissimilarity. Each module contains a different gene cluster and is marked with a different color.

Compared to STP, LTP demonstrated significantly enhanced postnatal growth rates and superior meat production. To identify genes strongly associated with the three key developmental stages in LTP, we conducted WGCNA we performed WGCNA using both breed and developmental time as phenotypic traits. Based on the gene expression data from the LD muscle across three developmental stages of LTP and STP, a filtered gene expression matrix comprising 8,958 genes was obtained. As illustrated in Figure 6A, the optimal soft-thresholding power (β = 9) was selected to construct an approximately scale-free topological overlap matrix. As shown in Figure 6B, the genes were clustered into 23 distinct co-expression modules. Among these, the darkred module contained the largest number of genes (961 genes), while the orange module had the fewest (55 genes) (Supplementary Table S3).

**Figure 7:**
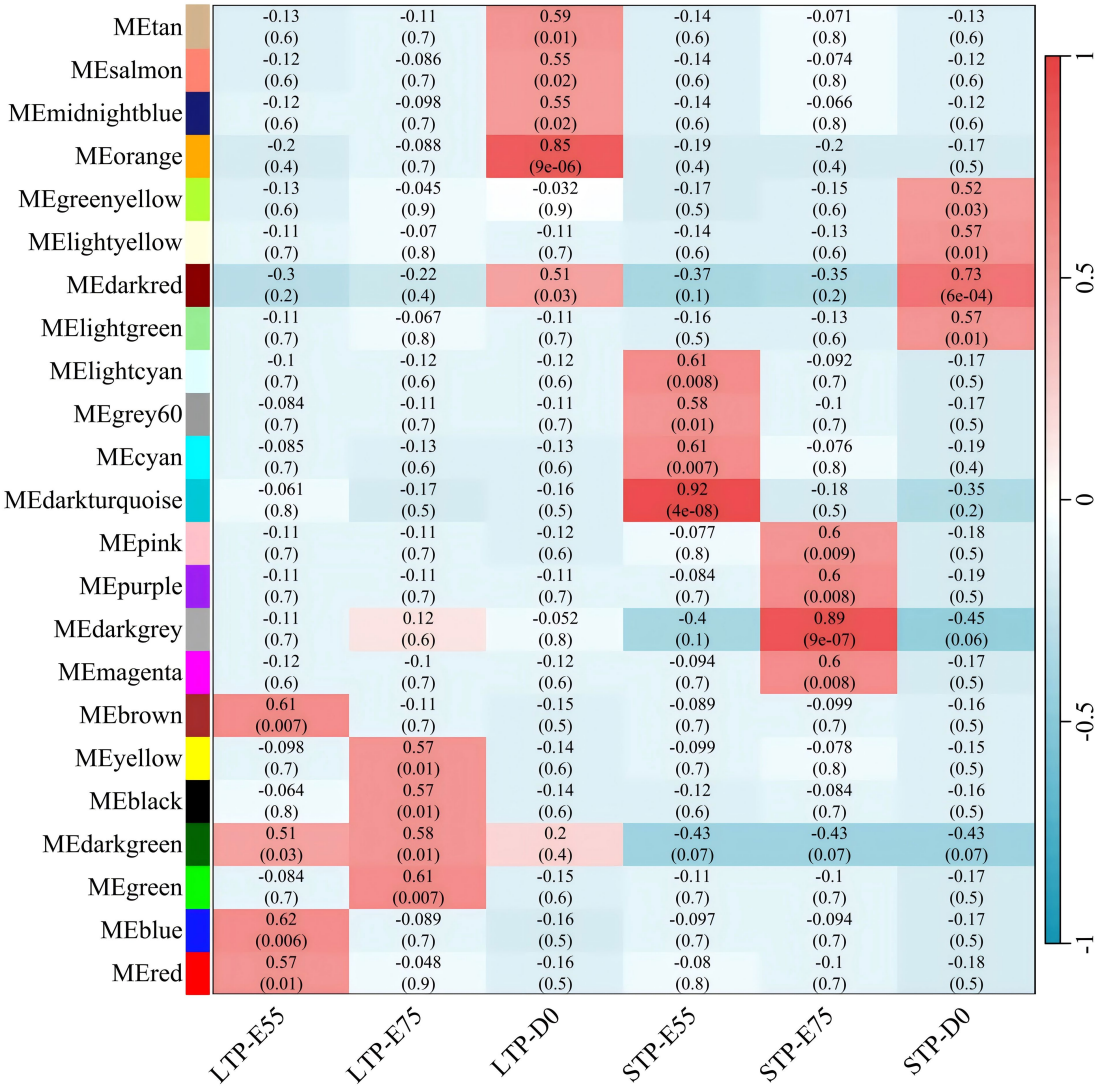
Module-trait relationships in large and small Diqing Tibetan pigs across three developmental stages (E55, E75, and D0). Abscissa is the trait, the ordinate is the module, the number of each grid represents the correlation between the module and the trait, and the number in parentheses represents *p*-value, red represents positive correlation and green represents negative correlation.

As depicted in Figure 7, the module-trait relationships analysis identified four co-expression modules (brown, darkgreen, blue, and red) significantly associated with LTP-E55, encompassing a total of 2,124 genes; four co-expression modules (yellow, black, darkgreen, and green) were found to be significantly correlated with LTP-E75, comprising 1,994 genes; five co-expression modules (tan, salmon, midnightblue, orange, and darkred) showed significant associations with LTP-D0, containing 2,017 genes. All these significant modules exhibited positive correlations with their respective traits, suggesting that the genes within these modules may play crucial roles in muscle development during the E55, E75, and D0 stages of LTP.

#### 3.3.2 Hub genes significantly positively correlated with three developmental stages of LTP

**Figure 8:**
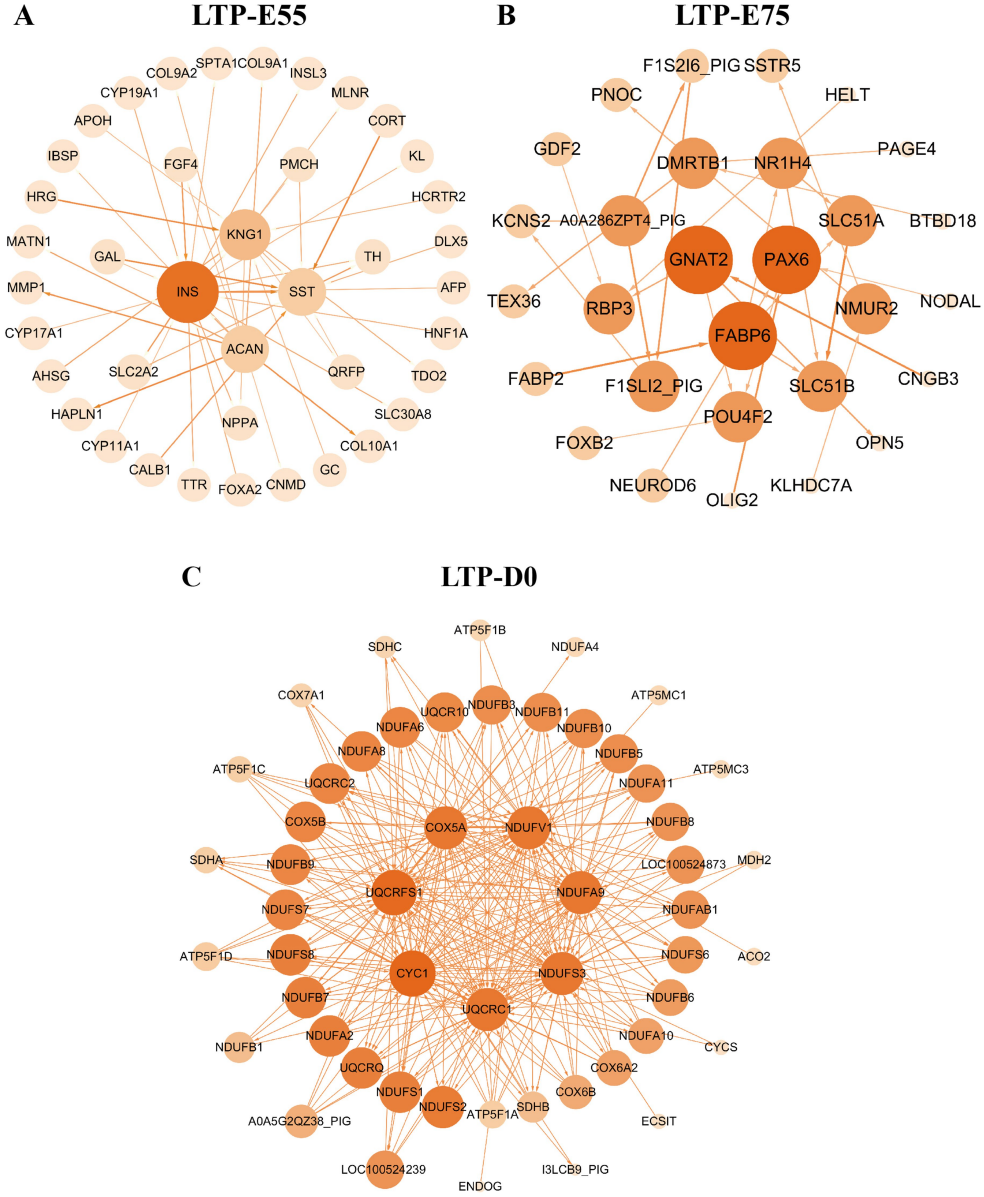
The interaction network of co-expression module genes significantly associated with three developmental stages of large Diqing Tibetan pigs. **(A)** The interaction network of LTP-E55. **(B)** The interaction network of LTP-E75. **(C)** The interaction network of LTP-D0. The intensity of the color corresponds to the degree of regulation.

Higher KME values indicate a stronger gene-module relationship (Supplementary Table S4). A total of 1,103 genes with KME values greater than 0.8 were identified from the modules significantly positively correlated with LTP-E55, with core genes including *INS*, *KNG1*, *SST*, and *ACAN*. From the modules significantly positively correlated with LTP-E75, 1,047 genes were identified, featuring core genes such as *GNAT2*, *PAX6*, and *FABP6*. Additionally, 1,181 genes were identified from the modules significantly positively correlated with LTP-D0, including core genes like *CYC1*, *UQCRFS1*, *COX5A*, *NDUFV1*, *NDUFA9*, *NDUFS3*, and *UQCRC1* (Figures 8A–C).

### 3.4 Verification of DEGs by real-time quantitative PCR

**Figure 9:**
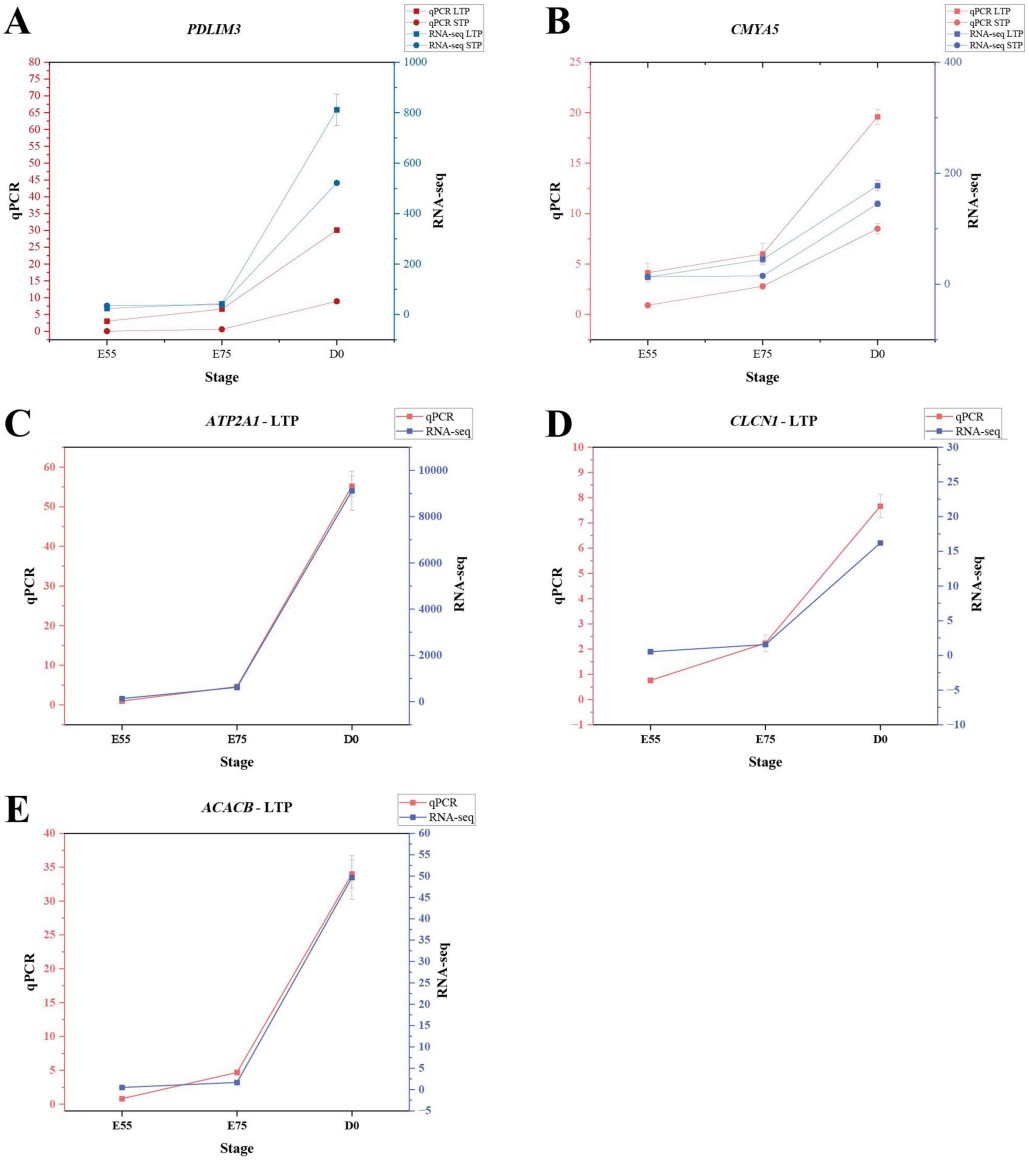
qPCR results of DEGs. **(A)** qPCR validation of PDLIM3 expression. **(B)** qPCR validation of CMYA5 expression. **(C)** qPCR validation of ATP2A1 expression in LTP. **(D)** qPCR validation of CLCN1 expression in LTP. **(E)** qPCR validation of ACACB expression in LTP.

To validate the RNA-seq results, qPCR was conducted on *PDLIM3*, *CMYA5*, *ATP2A1*, *ACACB* and *CLCN1*. As illustrated in Figure 9, the qPCR results displayed trends aligned with the RNA-seq data, thereby confirming the accuracy and reliability of the RNA-seq findings.

## 4 Discussion

### 4.1 A coordinated program of *CAPN6*, *STMN1*, and *ATP2A1* expression enhances secondary myofiber formation in LTP

In myoblasts, overexpression of *CAPN6* (Calpain 6) suppresses autophagy and inhibits myoblast differentiation and regeneration by sustaining mTOR signaling pathway activity (45). During secondary myofiber formation in LTP, numerous myoblasts fuse adjacent to primary myofibers to form new myotubes. The observed downregulation of *CAPN6* expression (Figure 3B) may therefore enhance secondary myofiber formation by promoting myoblast differentiation. *STMN1* (Stathmin 1), a microtubule-destabilizing protein, has been shown in multiple studies to promote cell proliferation (46, 47). We propose that decreased *STMN1* expression (Figure 3B) may drive LTP secondary myofiber formation by reducing myoblast proliferative capacity and facilitating the transition toward differentiation. *ATP2A1* (ATPase sarcoplasmic/endoplasmic reticulum Ca^2+^ transporting 1) is predominantly highly expressed in fast-twitch fibers (Figure 3B) (48, 49). Given that secondary myofibers exhibit fast-twitch characteristics (50), the progressively increasing expression of *ATP2A1* in LTP muscle suggests a potential correlation with the expanding population of secondary myofibers.

*CDK1* (Cyclin-dependent kinase 1), a core regulator of the cell cycle, promotes myoblast proliferation when highly expressed (51, 52). *SKA3* (Spindle and kinetochore-associated complex subunit 3) is a microtubule-binding component of the outer kinetochore and plays a critical role in cell division; studies indicate that *SKA3* expression drives cellular proliferation (53, 54). *ESPL1* (Extra spindle pole bodies like 1) functions primarily in initiating the final separation of sister chromatids, thereby sustaining cell cycle progression (55). *KIF11* (Kinesin family member 11), a motor protein involved in spindle formation and chromosome segregation (56), has been widely reported to promote cell proliferation (57, 58). *KIFC1* (Kinesin family member C1) contributes to centrosome integrity (59), and its knockdown has been shown to suppress cancer cell proliferation (60). *ANLN* (Anillin) encodes a key regulator of cytokinesis that acts as a scaffold protein to facilitate RhoA pathway activation and contractile ring assembly, ensuring successful cell division (61, 62); *ANLN* overexpression promotes proliferation in cancer cells (63). *KIF4A* (Kinesin family member 4A) is essential for proper chromosome segregation (64), and multiple studies demonstrate that its overexpression significantly enhances cell proliferation (65, 66). *CIT* (Citron rho-interacting serine/threonine kinase) regulates critical steps in cytokinesis, and its overexpression promotes cancer cell proliferation (67). Under physiological conditions, loss of *CIT* leads to cytokinesis failure and impaired proliferation (68). Upregulation of *CKAP2* (Cytoskeleton-associated protein 2) has been shown to directly stimulate cell proliferation in multiple cancer types (69, 70). *KIF20A* (Kinesin family member 20A), a kinesin superfamily protein, functions primarily during mitosis by participating in cell cycle regulation, microtubule dynamics, and cytokinesis; its overexpression enhances tumor cell proliferation (71, 72). *ZWINT* (ZW10 interacting kinetochore protein) plays an essential role in maintaining genomic stability, and its suppression significantly reduces proliferative capacity (73). *WEE1* (WEE1 G2 checkpoint kinase) expression facilitates cell cycle progression, while its inhibition suppresses cancer cell proliferation both *in vitro* and *in vivo* (74, 75). *CHEK1* (Checkpoint kinase 1) encodes a checkpoint kinase involved in DNA damage response, cell cycle control, and cell survival pathways. Its overexpression, primarily studied in cancer models, significantly promotes cell proliferation (76). During myofiber formation in STP, the progressive downregulation of the aforementioned genes (Figure 5B) suggests a potential shift in myoblasts from a proliferation-dominant state toward a differentiation phase, a trend consistent with observations in LTP. During embryonic muscle development in pigs, the early stage is primarily characterized by cell proliferation, followed by a transition to differentiation and fiber formation (77, 78). Thus, from E55 to D0, skeletal muscle development in both STP and LTP likely exits the rapid myoblast proliferation stage and enters a differentiation-dominant phase.

In STP, we also found that the expression level of *MYF5* gradually decreased with developmental time, while the expression levels of *MEF2C* and *TNNT1* continued to increase. *MYF5* (Myogenic factor 5) regulates the differentiation process of myogenic cells, and its enhanced expression promotes myogenic differentiation and functional improvement (79). The decreased expression of *MYF5* (Figure 5C) during myofiber formation in STP may consequently impair the capacity for secondary myofiber formation. *MEF2C* (Myocyte enhancer factor 2C) plays a pivotal role in regulating the formation and function of oxidative muscle fibers, facilitating the transition from glycolytic to oxidative fiber types (80, 81). *TNNT1* (Troponin T1, slow skeletal type) is a key regulatory factor in slow-twitch fiber development, involved in myofibril assembly, determination of contractile properties, and regulation of energy metabolism (82). During the embryonic period, primary myofibers are structurally predisposed to express slow-twitch-specific proteins (83), the continuous upregulation of *MEF2C* and *TNNT1* (Figure 5C) suggests a gradual enhancement in primary myofiber formation capability and an increasing abundance of primary myofibers in STP. These gene expression trends indicate that from E55 to D0, muscle development in STP appears to be primarily oriented toward the generation of primary myofibers. Primary myofibers form during the early embryonic stages, while secondary myofibers develop at a later phase; the combined number of both determines the total myofiber count in postnatal individuals (84). Secondary myofibers serve as the main source of muscle mass increase, and a higher proportion of secondary myofibers is associated with greater meat yield (85–87). Integrating these findings with previous results from LTP, we propose that LTP likely possesses a stronger capacity for secondary myofiber generation compared to STP, which may contribute to its higher postnatal meat yield.

### 4.2 Developmental stage-specific gene expression profiles underlie enhanced myogenesis in LTP

*INS* promotes skeletal muscle protein synthesis by activating the PI3K/AKT/mTOR signaling pathway (88) and can also upregulate the activity of glucose transporters in muscle cells enhancing glucose uptake efficiency to support energy metabolism (89). *KNG1* is known to inhibit the proliferation of gliomas (90) and mediates pro-inflammatory responses apoptosis and the generation of reactive oxygen species by activating the bradykinin system thereby influencing oxidative stress levels in livestock and poultry (91). *ACAN* as a core proteoglycan in cartilage regulates skeletal growth (92, 93) and may indirectly affect muscle development through the growth hormone signaling pathway (94). *SST* plays a crucial role in growth regulation and metabolism with maternal gene knockout leading to metabolic disorders and obesity in offspring (95). The expression of the aforementioned genes shows a significant positive correlation with LTP-E55, indicating that LTP at E55 may undergo enhanced energy metabolism to meet the energy requirements for rapid muscle development.

*PAX6* may promote the growth and differentiation of LTP muscle cells by participating in the regulation of the MAPK signaling pathway (96). *FABP6* interacts with the *KLF5* transcription factor, which may regulate muscle energy metabolism and cell proliferation by influencing cellular proliferation and lipid storage and utilization in muscle cells (97). *GNAT2* is primarily associated with light signal transduction in retinal cone cells (98), and currently, no studies have been found that directly investigate the relationship between *GNAT2* and skeletal muscle development. The significant positive correlation between the expression of *PAX6*, *FABP6*, and *KLF5* with LTP-E75 indicates that LTP at the E75 stage may undergo enhanced myocyte proliferation and differentiation.

*CYC1*, *UQCRFS1*, and *NDUFS3* are critical components of the mitochondrial electron transport chain and energy metabolism (96, 99–101). Their significant positive correlation with LTP-D0 indicates that LTP at the D0 stage may undergo enhanced energy metabolism to meet the energy demands required for rapid muscle development in LTP.

## 5 Conclusion

Through transcriptomic analysis of embryonic muscle tissue from Diqing Tibetan pigs exhibiting significant size variation within the same breed, we discovered that the higher postnatal meat yield in larger individuals may be attributed to their enhanced secondary myofiber formation capacity during embryonic development. Specifically, the developmental downregulation of *CAPN6* and *STMN1* expression promoted myoblast differentiation, while the concurrent upregulation of *ATP2A1* expression further facilitated secondary myofiber formation, collectively enhancing the secondary myofiber generative potential in large Diqing Tibetan pigs. Collectively, our findings provide a substantial theoretical foundation for genetic improvement and strategic utilization of local pig breeds.

## Data Availability

The original contributions presented in the study are publicly available. This data can be found here: National Center for Biotechnology Information (NCBI) BioProject, https://www.ncbi.nlm.nih.gov/bioproject/, PRJNA1228663.

## Footnotes

^1^ http://bioinfo.org/kobas/

^2^ http://string-db.org/

^3^ http://www.ncbi.nlm.nih.gov/tool/primer-blast/
